# Rifabutin-Containing Triple Therapy (RHB-105) for Eradication of *Helicobacter pylori*: Randomized ERADICATE Hp Trial

**DOI:** 10.3390/antibiotics9100685

**Published:** 2020-10-09

**Authors:** Ira N. Kalfus, David Y. Graham, Dennis S. Riff, Raymond M. Panas

**Affiliations:** 1 Independent Consultant, M2g Consulting, 251 Central Park West, New York, NY 10024, USA; 2 Department of Medicine, Michael E. DeBakey VA Medical Center and Baylor College of Medicine, 2002 Holcombe Boulevard, Houston, TX 77030, USA; dgraham@bcm.edu; 3 Anaheim Clinical Trials, 1085 N. Harbor Blvd, Anaheim, CA 92801, USA; dsriff@aol.com; 4 Medical Affairs, RedHill Biopharma, Inc., 8045 Arco Corporate Drive, Suite 200, Raleigh, NC 27617, USA; raypanas@gmail.com

**Keywords:** RHB-105, rifabutin, *Helicobacter pylori*, standard-of-care, dyspepsia, clinical trial

## Abstract

Due to increasing resistance to commonly used antibiotics, the World Health Organization and Food and Drug Administration have advocated the development of new therapeutic regimens for *Helicobacter pylori* (*H. pylori*). This phase three, double-blind study (ERADICATE Hp) randomized (2:1) treatment-naïve adults with *H. pylori* infection and dyspepsia to RHB-105 (an all-in-one combination of omeprazole 40 mg, amoxicillin 1000 mg, and rifabutin 50 mg) or an identically-appearing placebo, both administered every 8 h for 14 days. The *H. pylori* eradication rate with RHB-105, using a modified intent-to-treat (mITT) population of subjects who received ≥1 dose of study drug and had test-of-eradication performed 28–35 days post-completion of therapy, was compared (one-sample *Z*-test) to a literature-derived comparator rate of 70% and success rate with physician-selected standard-of-care given to placebo failures. The mITT *H. pylori* eradication rate (95% CI) with RHB-105 of 89.4% (82.0–96.8%) was greater than both the literature-derived comparator rate (*P* < 0.001) and the standard-of-care rate of 63.0% (44.8–81.1%) (*P* = 0.006). Adverse events with an incidence ≥5% for RHB-105 were diarrhea (12.7%), headache (11.9%), chromaturia (9.3%), abdominal tenderness (6.8%), and dizziness (5.1%). No leukopenia was noted. RHB-105 (Talicia^®^) proved to be a safe and effective empiric therapy for *H. pylori* eradication.

## 1. Introduction

*Helicobacter pylori* (*H. pylori*) is recognized as the primary etiologic agent responsible for gastritis, peptic ulcers, and gastric cancer [1,2]. Importantly, *H. pylori* is classified as a Group 1 carcinogen by the International Agency for Research on Cancer [3] and is considered the primary cause of human gastric cancer. A recent analysis of over 370,000 health records from the Veteran’s Administration database showed that eradication of *H. pylori* from patients with confirmed infection resulted in a 75% reduction in the risk of developing gastric cancer [4]. The World Health Organization (WHO) estimates that *H. pylori* is present in about half of the world’s population, while the Centers for Disease Control and Prevention (CDC) estimates that 30–40% of Americans harbor the infection [5,6,7,8]. Specifically, in the United States, the prevalence by race is higher in Hispanics (60%) and African Americans (54%) compared to White non-Hispanics (20%) [9]. While there is a similarity in infection rates by gender, age over 60 years presents a higher risk of infection (50%) compared to those under 30 years (20%) [9].

The clinical utility of current treatment guidelines for eradication of *H. pylori* infections has steadily declined due to the growing resistance to commonly prescribed antibiotic therapies [10,11]. *H. pylori* antibiotic resistance, particularly to clarithromycin, metronidazole, and levofloxacin, is now a major concern among clinicians [1,11,12,13]. This recent decline in therapeutic efficacy is the key rationale for why the WHO and United States (US) Food and Drug Administration (FDA) is targeting *H. pylori* for the development of new therapeutic agents [10,14]. Typical regimens of treatment currently include triple therapy (e.g., a proton pump inhibitor (PPI) with amoxicillin and a second antibiotic) or a quadruple therapy (e.g., PPI with bismuth and two antibiotics) with treatment durations ranging from 7–14 days [1,12,13]. However, the effect of the worldwide increase in antimicrobial resistance, including those antibiotics empirically used to treat *H. pylori* infection, has led to calls for new antibiotic regimens to eradicate *H. pylori*.

The rifamycin derivative rifabutin is potentially an excellent option for *H. pylori* eradication as rifabutin (1) provides bactericidal activity against *H. pylori* [15,16,17,18,19,20], (2) achieves high intracellular and intragastric concentrations [21,22], and (3) resistance is rare even after failure to cure *H. pylori* [23]. Rifabutin resistance has generally been reported only after the administration of high doses for extended durations such as those required for the treatment of mycobacterial infections. Prior experience has shown that the risk of development of antimicrobial resistance with *H. pylori* is further reduced when it is used with another antimicrobial agent such as amoxicillin [24,25]. In addition, the development of rifabutin resistant to *H. pylori* in vitro has shown to be very low (estimated at one in 10^9^) [17].

A prior meta-analysis of 2982 subjects receiving rifabutin triple therapy for *H. pylori* eradication reported a mean eradication rate of 73% (range of 66–79%) despite one to four prior treatment failures with other regimens [23]. The recent American College of Gastroenterology (ACG) Clinical Guideline for the treatment of *H. pylori* infection also recommends high dose rifabutin (300 mg once daily dose) as salvage therapy for those who fail multiple courses of *H. pylori* therapy [11]. However, Borody et al. studied a lower dose rifabutin regimen that provided high cure rates in 130 subjects who had failed standard clarithromycin-based triple therapy [26]. They achieved an overall eradication rate of 90.8%, and resistance to metronidazole or clarithromycin did not reduce the *H. pylori* eradication rate [26]. Based on this experience, RedHill Biopharma designed and implemented the ERADICATE Hp study (NCT01980095) to assess the safety and efficacy of RHB-105, an all-in-one capsule containing a fixed-dose combination of omeprazole (120 mg/day), amoxicillin (3000 mg/day), and rifabutin (150 mg/day) in patients with proven *H. pylori* infections. Results of the double-blind, placebo-controlled ERADICATE Hp study demonstrate that RHB-105, an all-in-one rifabutin triple therapy, is a safe and effective empiric therapy for *H. pylori* eradication.

## 2. Study Results

### 2.1. Participants

**Figure 1:**
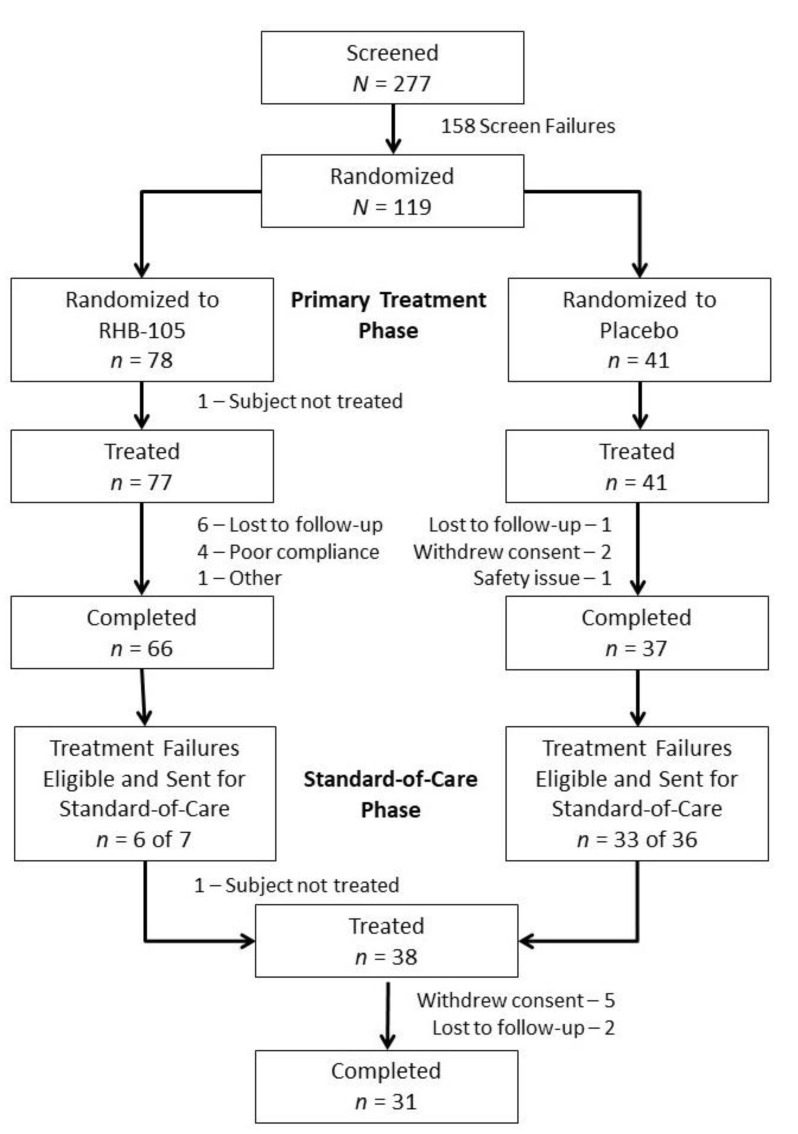
Consolidated Standards of Reporting Trials (CONSORT) Diagram.

Overall, 277 subjects were screened, 119 subjects were randomized, and 118 subjects received at least one dose of study drug (77 and 41 in the RHB-105 and placebo groups, respectively) (Figure 1). The treatment groups were similar, in general, based on demographics (Table 1). Subjects were primarily female (62.7%) and White (92.4%); their average age was 46.0 years. More subjects in the placebo group were Hispanic (85.4% vs. 77.9% of subjects in the RHB-105 group). Ninety-three subjects (78.8%) completed the study, and 25 (21.2%) discontinued prematurely.

Forty-three treatment failures (7 and 36 subjects in the RHB-105 and placebo groups, respectively) were eligible to be treated in the standard-of-care phase (Figure 1); 39 subjects chose to participate, and 38 received physician-selected standard-of-care. Three of the seven treatment failures in the RHB-105 group agreed to undergo endoscopy; all three strains of *H. pylori* isolated were susceptible to amoxicillin and clarithromycin, and two of three were susceptible to metronidazole. During this second phase of the study, 31 subjects completed standard-of-care treatment and underwent follow-up test-of-eradication to determine *H. pylori* eradication status.

### 2.2. Treatment Efficacy Results

For the primary endpoint (modified intent-to-treat (mITT)), the *H. pylori* eradication rate (95% CI) with RHB-105 was 89.4% (82.0–96.8%) (Table 2), which was significantly greater (*P* < 0.001) than the literature-derived historical comparator rate of 70%. The *H. pylori* eradication rate was statistically significant (*P* < 0.001) for all sites (or cluster of sites that enrolled four or fewer subjects) with the exception of two sites (66.7% (*n* = 3), *P* = 0.549; 81.8% (*n* = 22), *P* = 0.075). In a sensitivity analysis based on the intent-to-treat (ITT) population, the between-group difference (*H. pylori* eradication rate of 76.6% (vs. 70%; *P* = 0.085)) did not reach the level of statistical significance which was greatly influenced by 11 subjects failing to return for ^13^C urea breath test (UBT) at the test-of-eradication visit. The results of the sensitivity analysis in the per-protocol (PP) population (*H. pylori* eradication rate of 88.9% (vs. 70%; *P* < 0.001)) was similar to the primary analysis. Among the placebo subjects, one subject was noted to be *H. pylori* negative at the test-of-eradication visit (eradication rate: 2.4% (1/41)—ITT; 2.7% (1/37)—mITT; 2.8% (1/36)—PP). While the *H. pylori* eradication rate with RHB-105 was significantly greater compared to that of placebo (*P* < 0.001), this evaluation was not considered relevant or informative for efficacy.

### 2.3. CYP2C19 Genotyping

In the mITT population, 65 subjects receiving RHB-105 had blood samples for CYP2C19 status tested. Based on CYP2C19 genotypes, there were 11 rapid, 38 normal, 16 intermediate, and no ultra-rapid or poor metabolizers. No statistically significant difference was observed (*P* = 0.123) between the *H. pylori* eradication and failure groups based on the CYP2C19 status (while assessing the distribution over the different genotypes) among subjects randomized to RHB-105.

### 2.4. Physician-Selected Standard-of-Care Efficacy Results

The *H. pylori* eradication rate with RHB-105 treatment during the double-blind phase was significantly greater than the rate following physician-selected standard-of-care of treatment-naïve placebo subjects (mITT: 89.4% vs 63.0%, *P* = 0.006; ITT: 76.6% vs 51.5%, *P* = 0.009; Table 2). While physicians could choose any standard-of-care therapy, a clarithromycin-containing triple regimen was the most commonly prescribed, for which the *H. pylori* eradication rate (mITT) was 60.9% (Table 3).

### 2.5. Safety Results

Headache and diarrhea were the most commonly reported adverse events (Table 4). Chromaturia was also reported but is a known effect associated with the use of rifabutin. Most reported adverse events were mild or moderate in severity. A severe treatment-emergent adverse event was reported for four subjects: rash and diarrhea for one RHB-105-treated subject each and anemia and perirectal abscess for one placebo-treated subject each; the latter event was the only treatment-emergent serious adverse event reported. Three subjects discontinued due to adverse events. Among the RHB-105 subjects, one subject reported moderate pharyngitis, and one subject reported mild nausea with vomiting. Among the placebo subjects, one subject reported severe diarrhea with nausea and vomiting. Only the nausea with vomiting experienced by a RHB-105-treated subject was considered possibly related to the study drug. Bone marrow suppression, which has been reported with long term rifabutin use, was absent in this study.

## 3. Discussion

The first approval of an *H. pylori* eradication therapy was nearly 25 years ago. No new therapies or combinations have been approved in the decade prior to the development of RHB-105. As such, there is a growing concern by agencies and organizations such as the WHO, FDA, ACG, and European Medicines Agency about the increasing lack of effective treatment options for *H. pylori* infection. Increasingly resistant *H. pylori* has been noted in the literature and confirmed by the findings from this study. Additionally, the general absence of knowledge about local resistance rates creates a challenge for physicians to treat *H. pylori* effectively and empirically. Together, this suggests the need for a new antibiotic treatment for eradicating *H. pylori* infections.

This double-blind, randomized, controlled trial investigated a rifabutin-containing triple therapy combining three drugs (rifabutin, amoxicillin, and omeprazole) into an all-in-one capsule. The *H. pylori* eradication rate with RHB-105 was statistically significantly greater than that achieved by the historical control in the protocol-defined mITT analysis. Pharmacogenetic testing suggested no significant findings based on the overall metabolism of RHB-105. The study also demonstrated the need for follow-up test-of-eradication assessment to verify eradication, as noted by the ITT population analysis. The evidence from this study supported the further development of RHB-105, which resulted in the conduct of a similar study that established rifabutin as a safe and effective first-line therapy for *H. pylori* infection [30]. Furthermore, among treatment failures given the option for physician-selected standard-of-care, clarithromycin-containing triple therapy was by far the first choice by most physicians, despite guidelines emphasizing issues regarding clarithromycin resistance [12,13]. RHB-105 was well-tolerated based upon comparison to placebo control, with diarrhea and headache being the most commonly reported adverse events. The results of EDADICATE-Hp and the companion phase three study EDADICATE-Hp2 [30] formed the basis for FDA approval of RHB-105 as first-line treatment for eradicating *H. pylori*.

Study limitations included potential for unblinding given chromaturia present with RHB-105. However, the hard endpoint of ^13^C UBT mitigates this concern. Placebo was not an ideal comparator given the lack of efficacy with placebo; however, a placebo-control was included for safety reporting purposes and at the suggestion of the FDA given the short course of therapy and the availability of physician-selected standard-of-care for treatment failures. Finally, 11 subjects in the ITT group failed to return for test-of-eradication, with the majority lost to follow-up. Further studies will focus more intensively on subject follow-up and retention.

## 4. Materials and Methods

The study was registered at ClinicalTrials.gov as NCT01980095.

### 4.1. Ethical Approval

All procedures performed in this study involving human participants were in accordance with the ethical standards of the institutional and/or national research committee and with the 1964 Helsinki declaration and its later amendments or comparable ethical standards.

### 4.2. Participant Eligibility

Treatment-naive adults (age 18–65 years) with dyspepsia at least two weeks in duration and who had active *H. pylori* infection—based on positive ^13^C urea breath test (UBT), confirmed by fecal antigen test or diagnosis by gastric biopsy within 12 weeks of screening or prior to randomization—were eligible for enrollment. Participants were required to use specific contraceptive methods and refrain from eating foods or taking medications known to affect CYP3A4 or CYP2C19 pathways. Participants were excluded from the study who had received prior *H. pylori* eradication therapy; used any antibiotics four weeks prior to screening; used any bismuth-containing medications (such as Pepto-Bismol) or PPIs within the two weeks immediately prior to screening; used prohibited medicines within s days of randomization; presented with ≥ three active gastric and/or duodenal ulcers, gastric outlet obstruction; had a history of esophageal or gastric surgery or gastric cancer within the past five years; had a positive screen for HIV or hepatitis B or C; were pregnant, lactating or, unwilling to use specified contraception; or were hypersensitive to the study drugs.

### 4.3. Study Design

ERADICATE Hp was a Phase three, randomized, double-blind, placebo-controlled study of RHB-105 in *H. pylori*-infected adults. The study was conducted between November 25 2013 and August 24 2015. Subjects were screened for study participation at 12 sites in the United States and enrolled at nine sites.

The study consisted of a screening period (Days −42 to Day 0) to assess subject eligibility for enrollment. Eligible participants were randomized (2:1), based on a computer-generated randomization schedule (random blocks of six and three, respectively, by site), to RHB-105 or placebo; double-blind treatment was initiated on Day one and continued for 14 days during the double-blind treatment phase. An initial test-of-eradication visit occurred 28–35 days post completion of double-blind treatment.

Those subjects who tested positive for *H. pylori* at the initial test-of-eradication visit were invited to enter a standard-of-care treatment phase. At the beginning of the standard-of-care phase, blinded reviewers determined the randomized treatment assignment of the subjects. Those randomized to RHB-105 were offered endoscopy and then physician-selected, susceptibility-based anti-*H. pylori* treatment. Those randomized to placebo were offered physician-selected anti-*H. pylori* therapy without endoscopy. Treatment during the standard-of-care phase was at the discretion of the treating physician and the subject. A second test-of-eradication was performed approximately 28–35 days post completion of therapy.

### 4.4. Study Drug and Concomitant Medications

The study drug consisted of RHB-105 all-in-one capsules containing 10 mg omeprazole, 250 mg amoxicillin, and 12.5 mg rifabutin (Talicia^®^, RedHill Biopharma Ltd., Raleigh, NC, USA). A matching placebo capsule was used as reference therapy. Subjects took four capsules of study drug (RHB-105) every 8 h for 14 days (total daily doses of amoxicillin = 3 g, omeprazole = 120 mg, and rifabutin = 150 mg).

Antibiotics, PPIs (other than that contained within the study drug), and bismuth-containing drugs were prohibited during treatment and post-treatment through the test-of-eradication visit. Histamine-2 receptor antagonists and antacids were prohibited 24 h prior to ^13^C UBT testing.

### 4.5. Efficacy and Safety Assessments

Patients underwent follow-up ^13^C UBT testing (BreathTek^®^, Otsuka America Pharmaceutical Inc., Rockville, MD) at the test-of-eradication visit to determine *H. pylori* eradication. Subjects in the RHB-105 group with positive ^13^C UBT results were offered endoscopy with culture and antibiotic susceptibility testing. Pharmacogenetic testing was performed on blood samples collected at baseline to assess CYP 2C19 status. Safety of study drug was assessed based on the occurrence and severity of adverse events during the study as well as changes from baseline in hematology, chemistry, and urinalysis laboratory values.

### 4.6. Statistical Methods

All participants who received at least one dose of randomized study drug were included in the safety analysis data set. Protocol-defined analyses of the primary endpoint were performed in a modified intent-to-treat (mITT) population, which included all subjects who received at least one dose of randomized study treatment and underwent ^13^C UBT test-of-eradication 28–35 days post-completion of therapy. The mITT population was selected to assess actual clinically relevant treatment eradication among the study subjects. Sensitivity analyses were performed in an intent-to-treat (ITT) population, in which subjects who were lost to treatment, dropped out of the study, or failed to return for test-of-eradication were imputed as treatment failures. Sensitivity analyses were also performed in a per-protocol (PP) population, which included all subjects who consumed at least 75% of planned study treatment received, underwent ^13^C UBT at the initial test-of-eradication visit, and did not have any major protocol violations.

#### 4.6.1. Efficacy Analysis

The primary endpoint was *H. pylori* eradication rate at the initial test-of-eradication visit, assessed in the mITT population. The *H. pylori* eradication rate among the RHB-105 subjects was compared to an estimated literature-derived comparator *H. pylori* eradication rate of 70% [27,28,29] using a one-sample Z-test. Sensitivity analysis by center was performed for the mITT population, with sites that enrolled four or fewer subjects combined for the analysis. Sensitivity analyses on the primary endpoint in the ITT and PP populations were also performed using a one-sample Z-test. A similar analysis was provided for the mITT and ITT population, assessing the eradication rates of physician-selected standard-of-care.

#### 4.6.2. Exploratory Efficacy Analyses

Due to the fact that omeprazole is metabolized by CYP 2C19, and rifabutin is a CYP inducer, pharmacogenetic testing was performed on blood samples collected at baseline to assess CYP 2C19 status. The primary endpoint was analyzed by CYP 2C19 genotype and phenotype (classified as ultra-rapid, rapid, normal, intermediate, and poor metabolizers). In addition, culture and sensitivity testing to rifabutin, amoxicillin, clarithromycin, and metronidazole was offered for all subjects randomized to RHB-105 who were treatment failures at the initial test-of-eradication visit (*n* = 7). Most (*n* = 4) chose not to have endoscopy; among the few that did (*n* = 3), results were generally not yet available to treating physicians prior to study completion and were generally not pursued by investigators or subjects. The collected samples were analyzed using a validated methodology and reported separately.

### 4.7. Data Sharing Statement

The study protocol, statistical analysis plan, informed consent form, and data collected for this study, including de-identified individual participant data and a data dictionary defining each field in the set, will be made available upon consideration and approval of RedHill Biopharma following the publication of this paper.

## 5. Conclusions

This study showed that RHB-105 provides both efficacious and well-tolerated treatment for the eradication of *H. pylori* infection. The use of an all-in-one rifabutin-containing triple therapy provides an option for overcoming current *H. pylori* treatment resistance and supports the use of rifabutin-containing triple therapy as an empiric first-line treatment.

## Figures and Tables

**Table 1:** Baseline subject demographics and characteristics.

Characteristic	RHB-105 *n* = 77	Placebo *n* = 41	Total *N* = 118
Age, y, mean ± SD	46.2 ± 10.56	45.8 ± 9.52	46.0 ± 10.18
Sex, n (%)			
Female	49 (63.6%)	25 (61.0%)	74 (62.7%)
Male	28 (36.4%)	16 (39.0%)	44 (37.3%)
Ethnicity, n (%)			
Hispanic or Latino	60 (77.9%)	35 (85.4%)	95 (80.5%)
Not Hispanic or Latino	17 (22.1%)	6 (14.6%)	23 (19.5%)
Race, n (%)			
White	71 (92.2%)	38 (92.7%)	109 (92.4%)
Black African heritage or African American	6 (7.8%)	3 (7.3%)	9 (7.6%)

**Table 2:** *Helicobacter pylori* eradication rate: RHB-105 compared to literature-derived historical control and physician-selected standard-of-care.

	ITT ^a^	mITT	PP
**RHB-105**	**76.6% (59/77)**	**89.4% (59/66)**	**88.9% (56/63)**
95% CI	67.2–86.1%	82.0–96.8%	81.1–96.7%
Literature-derived Historical Control ^b^	70%	70%	70%
*P*-value, RHB-105 vs. Literature-derived Historical Control	0.085	<0.001	<0.001
Physician-selected Standard-of-Care (Placebo Subjects)	51.5% (17/33)	63.0% (17/27)	NA
95% CI	34.5–68.6%	44.8–81.1%	
*P*-value, RHB-105 vs. Physician-selected Standard-of-Care	0.009	0.006	NA

ITT = intent-to-treat; mITT = modified intent-to-treat; NA = not applicable; PP = per-protocol. ^a^ Eleven of 77 subjects in the ITT population failed to return for test-of-eradication at visit four and were imputed as treatment failures. ^b^ Estimated *Helicobacter pylori* eradication rate of 70% derived from references [27,28,29]. Notes: The ITT population included all subjects who received at least one dose of randomized study treatment; those who were lost to treatment, dropped out of the study, or failed to return for test-of-eradication were imputed as treatment failures. The mITT population included all subjects who received at least 1 dose of randomized study treatment and underwent ^13^C urea breath test (UBT) test-of-eradication 28–35 days post-completion of therapy. The PP population included all subjects who consumed at least 75% of planned study treatment received, underwent ^13^C UBT at the initial test-of-eradication visit, and did not have any major protocol violations.

**Table 3:** *H. pylori* Eradication Rates with Physician-selected Standard-of-Care (mITT).

	Placebo	RHB-105 Treatment Failures	Total
All Subjects	17/27 (62.9%)	2/4 (50.0%)	19/31 (61.3%)
Clarithromycin triple therapy ^a^	14/23 (60.9%)	2/4 (50.0%)	16/27 (59.3%)
Metronidazole triple therapy ^b^	1/2 (50.0%)	0	1/2 (50.0%)
Bismuth quadruple therapy ^c^	2/2 (100.0%)	0	2/2 (100.0%)

mITT = modified intent-to-treat. ^a^ Clarithromycin, amoxicillin, and proton pump inhibitor (omeprazole or lansoprazole). ^b^ Metronidazole, bismuth subcitrate potassium, and tetracycline. ^c^ Bismuth subcitrate potassium, metronidazole, tetracycline, and omeprazole.

**Table 4:** Adverse Events Occurring in >2% of All Subjects and in a Greater Percentage of RHB-105 Subjects than Placebo Subjects.

Adverse Event	RHB-105 *n* = 77	Placebo *n* = 41	Total *N* = 118
All subjects with an adverse event	39 (50.6%)	19 (46.3%)	58 (49.2%)
Headache ^a^	12 (15.6%)	4 (9.8%)	16 (13.6%)
Diarrhea	11 (14.3%)	4 (9.8%)	15 (12.7%)
Chromaturia	10 (13.0%)	1 (2.4%)	11 (9.3%)
Rash	4 (5.2%)	0	4 (5.2%)
Nausea	3 (3.9%)	1 (2.4%)	4 (3.4%)
Oropharyngeal pain	3 (3.9%)	0	3 (2.5%)
Blood creatine phosphokinase increase	2 (2.6%)	1 (2.4%)	3 (2.5%)

^a^ Headache includes headache and migraine.
